# Supplementary figures and images for: Meningeal vascular Aβ deposition associates with cerebral hypoperfusion and compensatory collateral remodeling
Source: Alzheimers Res Ther. 2025 Nov 13;17:245. doi: 10.1186/s13195-025-01876-7 (PMC12616917; doi:10.1186/s13195-025-01876-7)

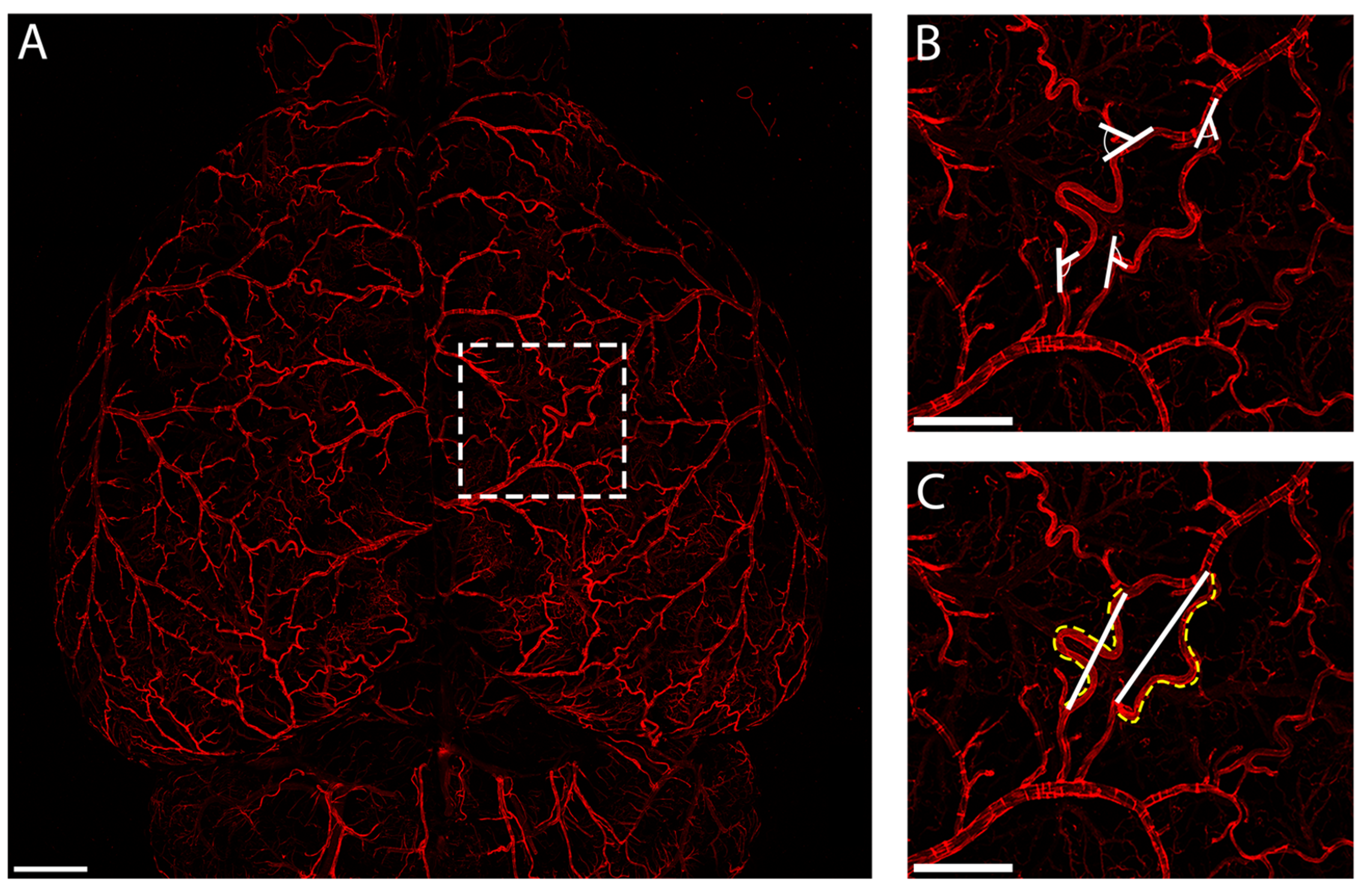

Supplement: Supplementary file 1 — Supplementary Material 1: Supplemental Fig. 1. Collateral identification from vessel painted images. (A) Representative tiled 4X confocal image of vessel painted male J20 brain. Collateral blood vessels are identified within the dashed box by their characteristic tortuosity and typically non-physiological, wide angles of attachment to the parent arteries. These intercollateral vessels, which connect two independent arteries, are positioned directly between the distal-most arterioles of the parent arteries. (B) Angle of attachment to the parent artery of collateral vessels. These vessels often exhibit wide, variable angles that can impact blood flow dynamics. (C) Collateral tortuosity is calculated using the straight line of collateral length (white) and freehand length (span; dashed yellow). The formula used is: tortuosity = freehand length/straight-line length X 100. Scale bars = 1 mm for panel A and 500 μm for panels B and C [file 13195_2025_1876_MOESM1_ESM.tif]

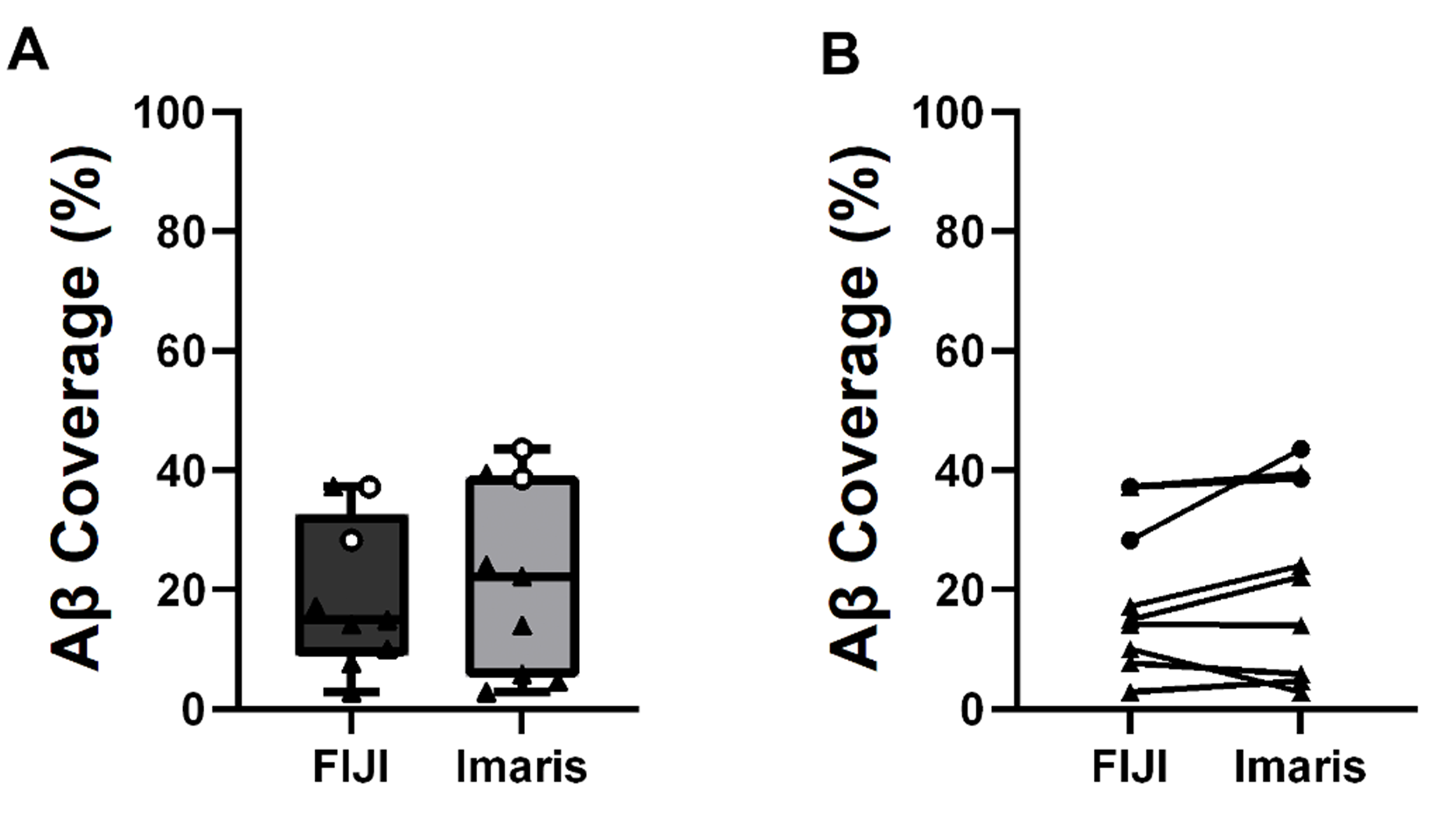

Supplement: Supplementary file 2 — Supplementary Material 2: Supplemental Fig. 2. Comparison of FIJI (ImageJ) and Imaris Aβ coverage analyses in 12-month-old J20 mice. (A) Average coverage of entire leptomeningeal vasculature by Aβ as quantified by ImageJ and Imaris analysis with (B) matched samples. No significance was detected. Significance tested for using unpaired (A) and paired (B) t-test. N = 7 female J20 and 2 male J20. Circles = males; triangles = females [file 13195_2025_1876_MOESM2_ESM.tif]

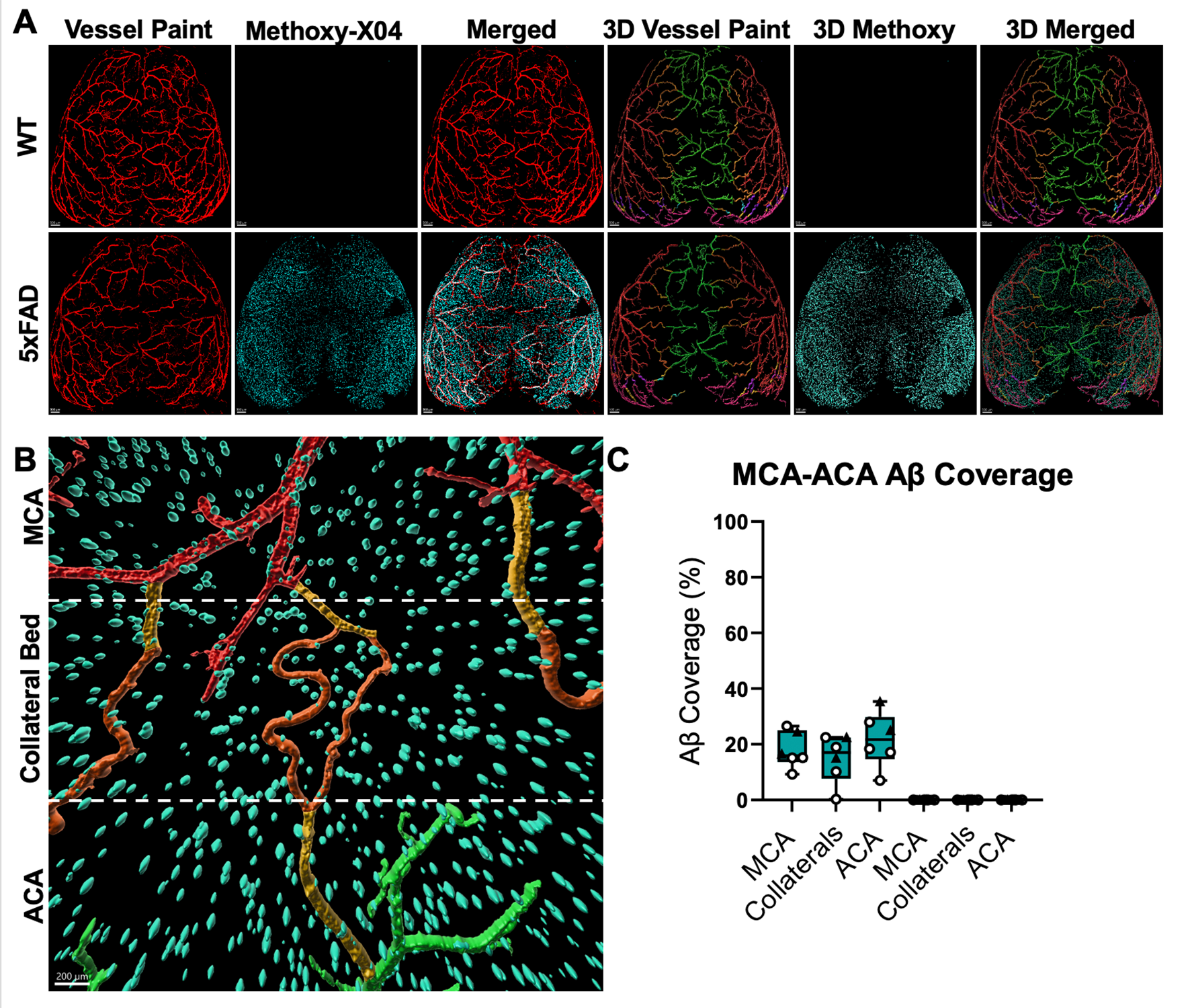

Supplement: Supplementary file 3 — Supplementary Material 3: Supplemental Fig. 3. Leptomeningeal Aβ coverage in the 12 month old male and female WT and 5XFAD mouse (A) Representative images of vessel paint (red) and Methoxy-XO4 (cyan) in 12-month-old WT and 5X FAD mice and corresponding 3D Imaris reconstructions (scale bar = 500 μm) (B) Representative zoomed image of MCA-ACA collateral bed in J20 animal (Anterior Cerebral Artery (ACA) = green, collaterals = orange, Medial Cerebral Artery (MCA) = red, distal arterials = gold, scale bar = 200 μm) (C) % Aβ coverage of the MCA, MCA-ACA Collaterals, and ACA with significance tested by RM One-Way ANOVA test. N = 4 male 5X FAD, 2 female 5X FAD, 4 male WT, and 5 female WT. Circles = males; triangles = females [file 13195_2025_1876_MOESM3_ESM.tif]

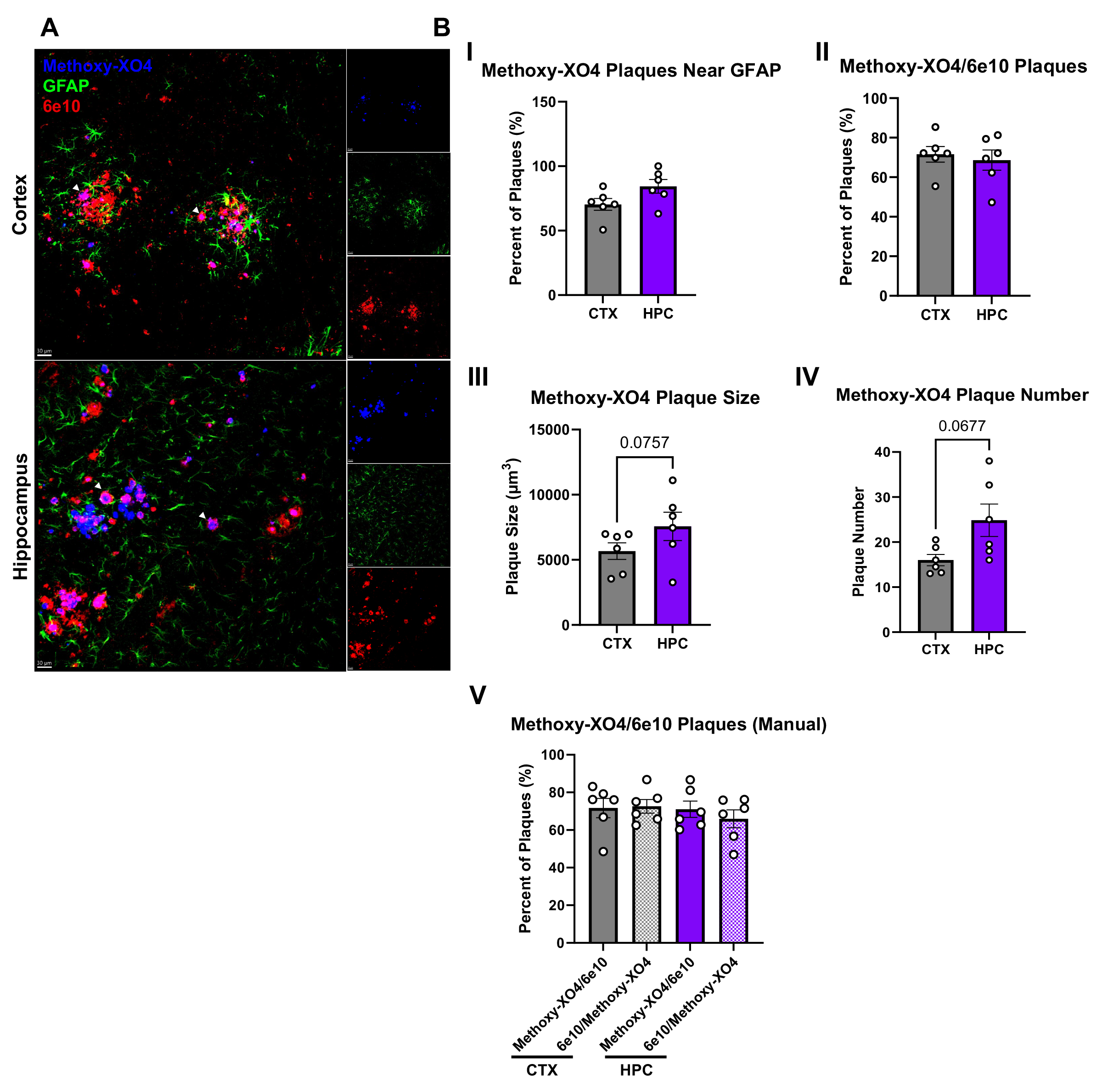

Supplement: Supplementary file 4 — Supplementary Material 4: Supplemental Fig. 4. Methoxy XO4 and antibody labeling approaches to identify parenchymal plaques (A) Representative images of Methoxy-XO4 (blue), GFAP (green) and 6e10 (red) in cortex and hippocampus of male J20 mice. GFAP staining was utilized to help confirm presence of plaques, which are known to induce glial reactivity. White arrows mark plaques which are co-labeled with Methoxy-XO4 and 6e10 (scale = 50 μm) (B) The number of Methoxy-XO4 labeled plaques within 10 μm of GFAP signal (II) The number of Methoxy-XO4 labeled plaques in contact with 6e10 signal (III) Methoxy-XO4 plaque size and (IV) number and (V) percentage of Methoxy-XO4 plaques that colabel with 6e10 and 6e10 plaques that colabel with Methoxy-XO4 in cortex and hippocampus. Significance was tested through paired t-test [file 13195_2025_1876_MOESM4_ESM.tif]

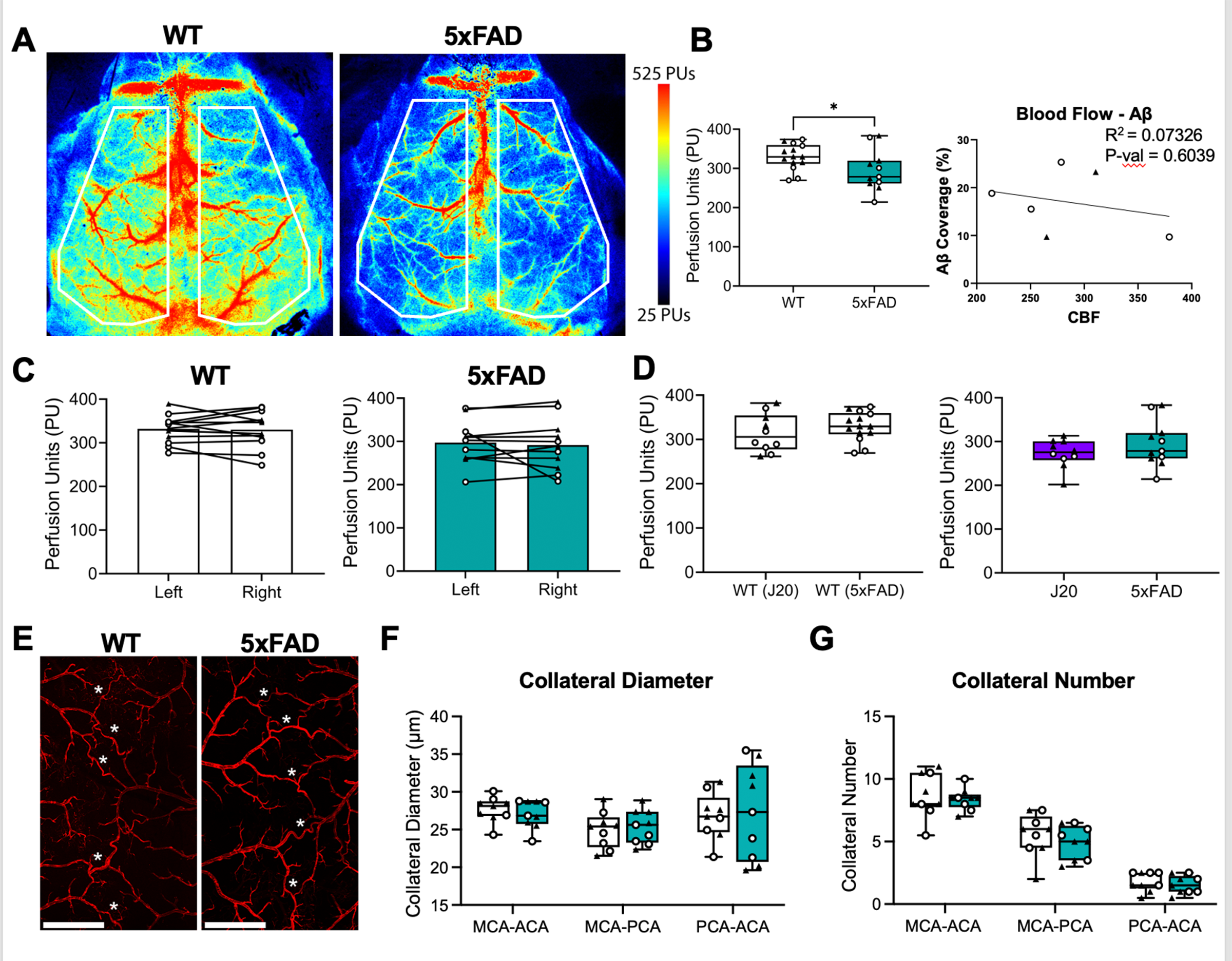

Supplement: Supplementary file 5 — Supplementary Material 5: Supplemental Fig. 5. Despite reduced blood flow, collateral diameter and collateral number is unaltered in aged 5XFAD mice. (A) Pseudocolored laser speckle contrast images of WT (n = 6 male, 8 female) and 5xFAD (n = 5 male, 6 female) mice at 12 months of age. (B) Analysis by unpaired t-test showed significant reduction in cerebral blood flow in 5xFAD mice compared to WT littermates. (C) No differences were seen in hemispheric perfusion (D) No significant differences were noted in cerebral blood flow between 5xFAD and J20 mice, or their control littermates. (E) Representative confocal images of the MCA-ACA pial collateral niche of WT and 5xFAD mice. (F) No differences were found in collateral diameter or (G) number between 5xFAD (n = 4 male and 5 female) and WT mice (n = 4 male and 5 female). Significant differences in collateral niches were evaluated using multiple unpaired t-tests. Circles = males; Triangles = females [file 13195_2025_1876_MOESM5_ESM.tif]

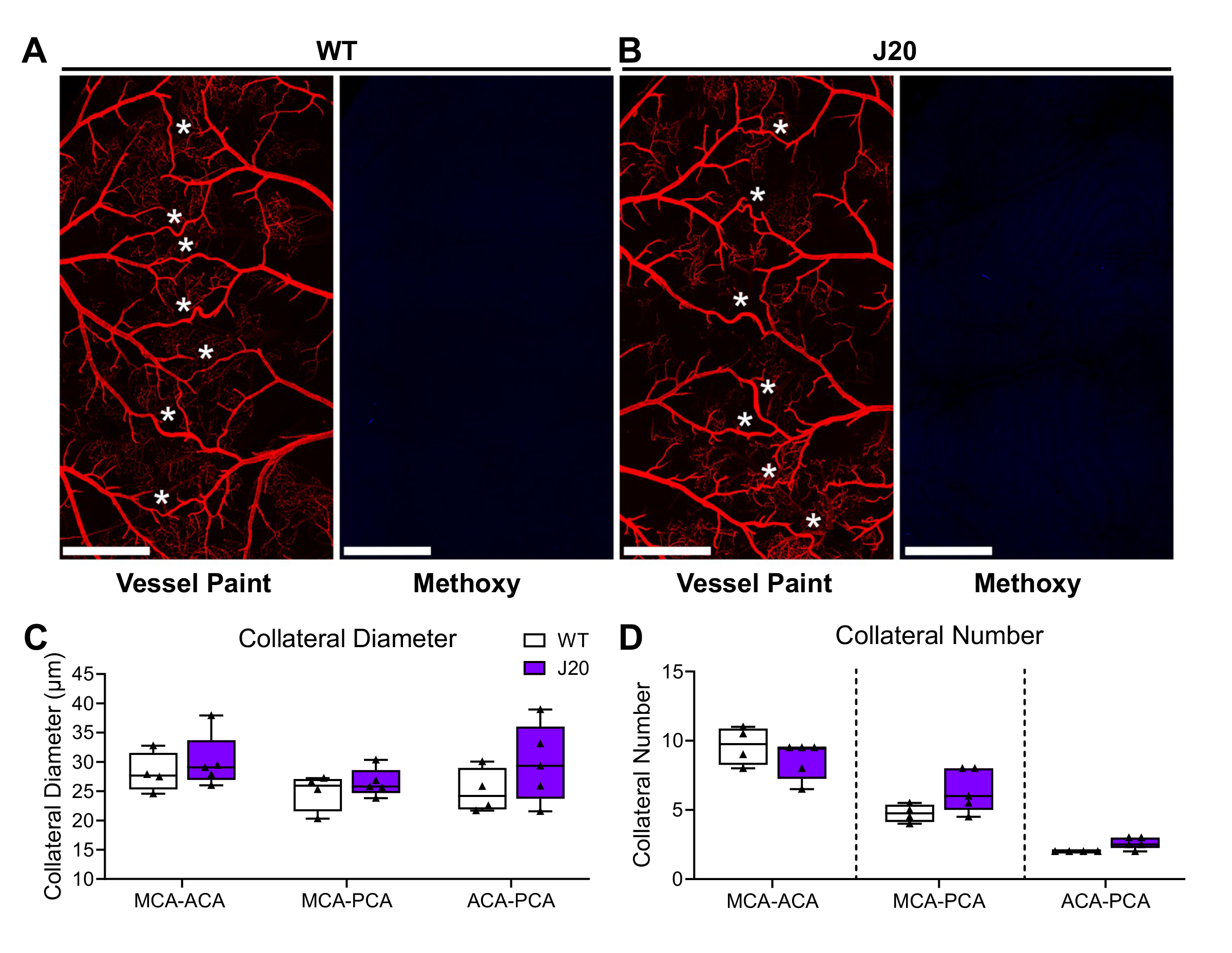

Supplement: Supplementary file 6 — Supplementary Material 6: Supplemental Fig. 6. Early life J20 mice do not show changes in collateral remodeling (A) Representative vessels painted and Methoxy-XO4 images of WT and (B) J20 mice at 2 months of age, prior to Aβ accumulation. (C) Collateral diameter and (D) number is unaltered in any of the intercollateral niches between WT (n = 5 female) and J20 (n = 5 female) mice. Significant differences in collateral niches were evaluated using multiple unpaired t-tests. White * = MCA-ACA collateral blood vessel. Scale bar = 1 mm. Significant differences in collateral niches were evaluated using multiple unpaired t-tests. Circles = males; Triangles = Females [file 13195_2025_1876_MOESM6_ESM.tif]

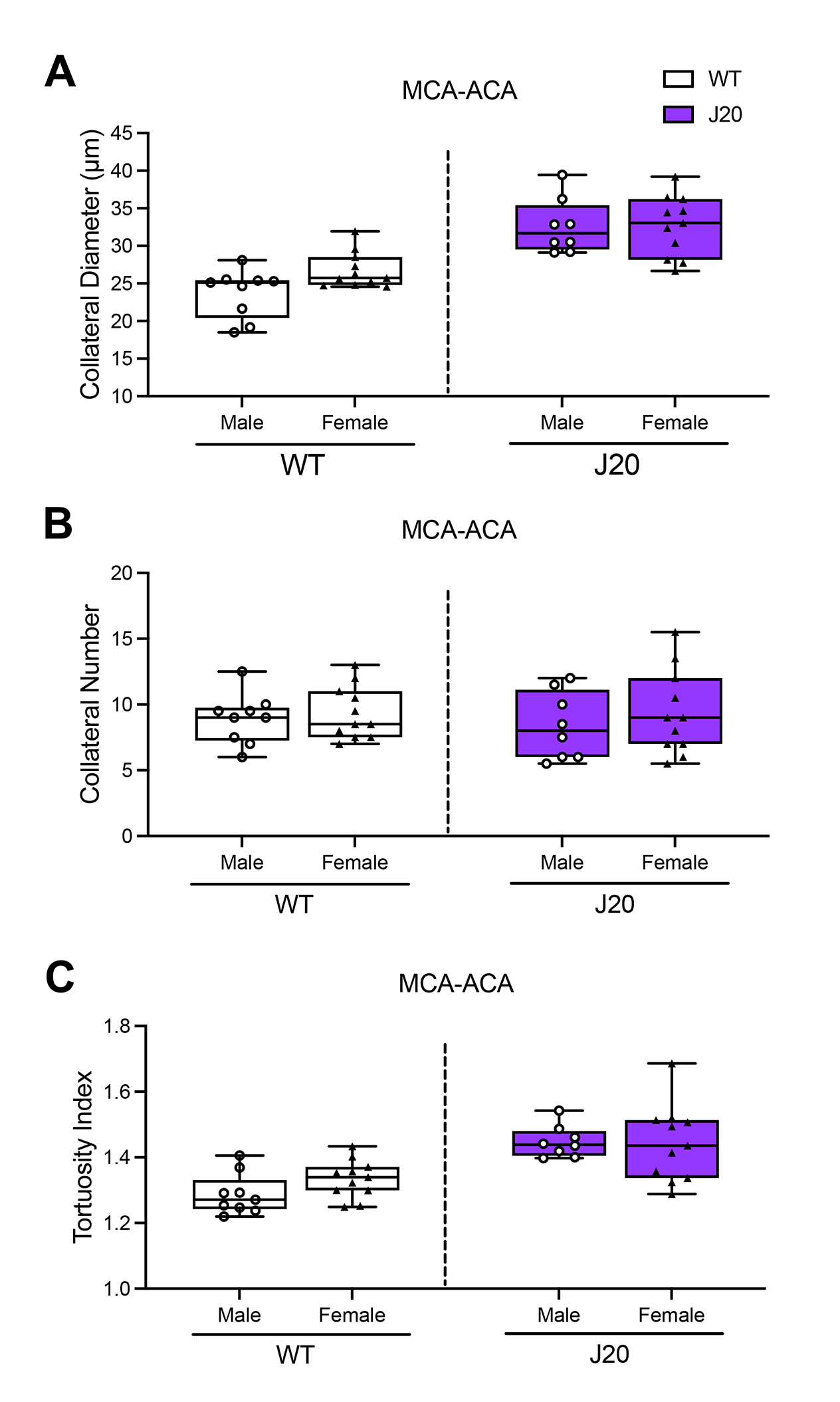

Supplement: Supplementary file 7 — Supplementary Material 7: Supplemental Fig. 7. Collateral characteristics do not differ between sexes. (A) No significant sex differences were noted in each genotype for collateral diameter, (B) number, (C) or tortuosity. Significant differences in collateral niches were evaluated using multiple unpaired t-tests. n = 9 male and 11 female WT, and 8 male and 11 female J20. Circles = males; Triangles = Females [file 13195_2025_1876_MOESM7_ESM.tif]

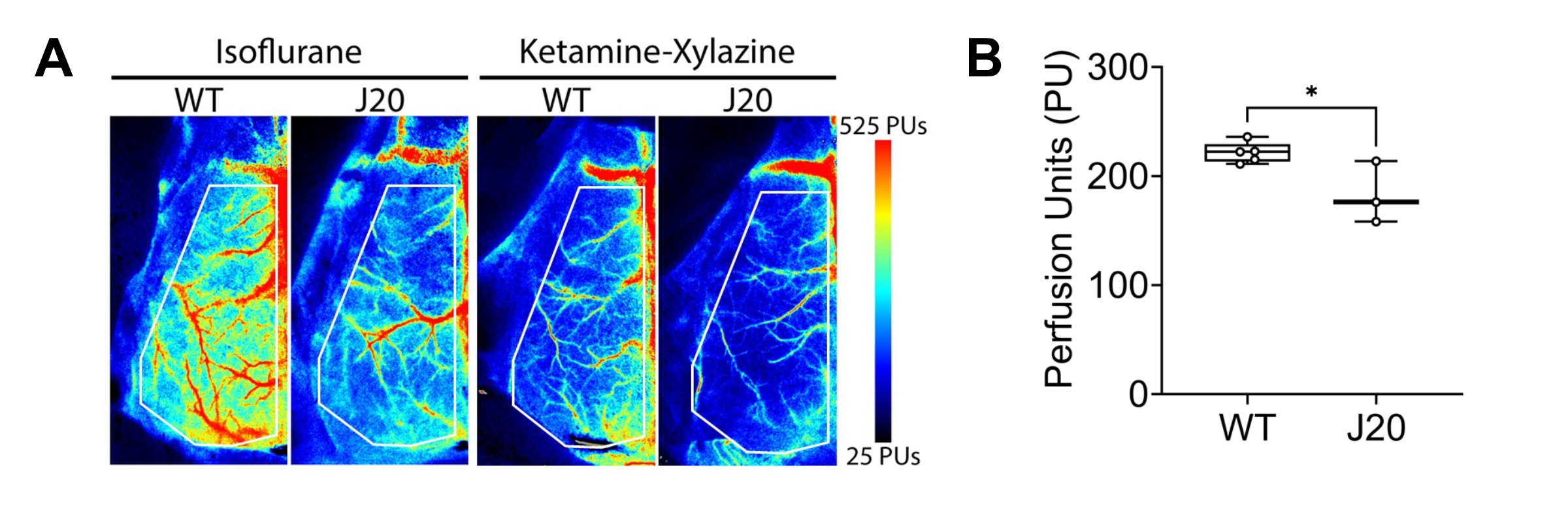

Supplement: Supplementary file 8 — Supplementary Material 8: Supplemental Fig. 8. Effect of anesthetic choice on cerebral perfusion measured by laser speckle contrast imaging. (A) Representative pseudocolored laser speckle contrast images of WT and J20 mice anesthetized with either isoflurane or ketamine/xylazine. (B) Quantitative analysis of averaged hemispheric perfusion demonstrates significantly reduced cerebral perfusion in J20 mice anesthetized with ketamine/xylazine. Statistical analysis was performed using an unpaired t-test. *p < 0.05, N = 3–5 males [file 13195_2025_1876_MOESM8_ESM.tif]
